# Heat shock protein 90 is downregulated in calcific aortic valve disease

**DOI:** 10.1186/s12872-019-01294-2

**Published:** 2019-12-19

**Authors:** Jonna Weisell, Pauli Ohukainen, Juha Näpänkangas, Steffen Ohlmeier, Ulrich Bergmann, Tuomas Peltonen, Panu Taskinen, Heikki Ruskoaho, Jaana Rysä

**Affiliations:** 1 School of Pharmacy, University of Eastern Finland, POB 1627, 70211 Kuopio, Finland; 2 Research Unit of Biomedicine, Computational Medicine, University of Oulu, Oulu, Finland; 3 Department of Pathology, Cancer Research and Translational Medicine Research Unit, University of Oulu and Oulu University Hospital, Oulu, Finland; 4 Proteomics and Mass Spectrometry Core Facilities, Biocenter Oulu, Faculty of Biochemistry and Molecular Medicine, University of Oulu, Oulu, Finland; 5 Department of Pharmacology and Toxicology, University of Oulu, Oulu, Finland; 6 Department of Cardiovascular Surgery, Oulu University Hospital, University of Oulu, Oulu, Finland; 7 Medical Research Center Oulu, Oulu University Hospital and University of Oulu, Oulu, Finland; 8 Drug Research Program and Division of Pharmacology and Pharmacotherapy, University of Helsinki, Helsinki, Finland

**Keywords:** Aortic valve stenosis, Calcified aortic valve disease, Heat-shock protein, Proteomics

## Abstract

**Background:**

Calcific aortic valve disease (CAVD) is an atheroinflammatory process; finally it leads to progressive calcification of the valve. There is no effective pharmacological treatment for CAVD and many of the underlying molecular mechanisms remain unknown. We conducted a proteomic study to reveal novel factors associated with CAVD.

**Methods:**

We compared aortic valves from patients undergoing valvular replacement surgery due to non-calcified aortic insufficiency (control group, *n* = 5) to a stenotic group (*n* = 7) using two-dimensional difference gel electrophoresis (2D-DIGE). Protein spots were identified with mass spectrometry. Western blot and immunohistochemistry were used to validate the results in a separate patient cohort and Ingenuity Pathway Analysis (IPA) was exploited to predict the regulatory network of CAVD.

**Results:**

We detected an upregulation of complement 9 (C9), serum amyloid P-component (APCS) and transgelin as well as downregulation of heat shock protein (HSP90), protein disulfide isomerase A3 (PDIA3), annexin A2 (ANXA2) and galectin-1 in patients with aortic valve stenosis. The decreased protein expression of HSP90 was confirmed with Western blot.

**Conclusions:**

We describe here a novel data set of proteomic changes associated with CAVD, including downregulation of the pro-inflammatory cytosolic protein, HSP90.

## Background

Calcific aortic valve disease (CAVD) is a progressive disease, which originates from endothelial cell damage on the aortic surface of aortic valve followed by an accumulation of oxidized lipids and the infiltration of inflammatory cells into the valve [1]. This promotes active remodeling of the extracellular matrix with the disorganization of collagen fibers, resulting in a thickening of the aortic valve leaflets. Furthermore, osteogenic programming of valve interstitial cells (VICs), causes progressive calcification and ultimately a severe obstruction of cardiac outflow. Several factors and signaling pathways have been linked to CAVD, e.g. interleukins, tumor necrosis factor (TNF), matrix metalloproteinases, bone morphogenic protein 2 (BMP2) and osteogenic regulator runt-related transcription factor 2 (RUNX2) [1, 2]. However, despite recent progress in understanding the molecular pathogenesis of CAVD, the factors driving the progression of this disease are not fully understood.

Various omics-analyses have been performed to gain a better understanding of the molecular mechanism underpinning CAVD. Transcriptomic studies of human CAVD have been undertaken to identify differentially expressed genes [3–6] and microRNAs [4, 7] in different stages of aortic valve calcification. In addition, there have been multiple proteomics studies of CAVD performed [8–16]. However, only three of them compared stenotic valves to control valves [9, 12, 16], and of these, only Schlotter et al. [12] reported the use of tricuspid valves in their study. Furthermore, Schlotter et al. [12] combined the results from proteomics and transcriptomics to describe the integrated molecular dataset of human CAVD [12].

In this study, we conducted a proteomic analysis of aortic valve calcification by comparing control and stenotic human aortic valves using two-dimensional difference gel electrophoresis (2D-DIGE). Selected proteomic changes were confirmed with Western blotting and immunohistochemistry. In addition, Ingenuity pathway analysis (IPA) was used to clarify the potential signaling pathways associated with identified proteins.

## Methods

### Patients

The aortic valves examined in this study were obtained from 50 patients at the time of aortic valve or aortic root surgery. All operations were made following normal surgical procedures. The study protocol was approved by the Research Ethics Committee of Oulu University Hospital and it conformed to the principles outlined in the Declaration of Helsinki. The aortic valve cusps were immersed immediately after removal into liquid nitrogen and stored at − 70 °C until analyzed.

For proteomics study, patients were divided into two groups: the control group (C, *n* = 5) consisted of patients with normal, non-calcified, smooth and pliable aortic valve cusps, operated due to ascending aortic pathology (aneurysm or dissection) or aortic regurgitation. The aortic stenosis group (AS, *n* = 7) consisted of patients who had non-rheumatic, severe aortic valve sclerosis with an increased degree of calcification. Patients who were identified as exhibiting macroscopic thickenings of aortic valve cusps, which were microscopically identified mainly as fibrotic and mild sclerotic lesions, were excluded from the study.

The patients’ demographics are presented in Table 1. There were no significant differences in gender, left ventricular ejection fraction or comorbidities between the study groups, and valve anatomy. However, the average age of the aortic stenosis (AS) patients was significantly higher than the patients in the control group. Histologically, the stenotic valves had a significantly elevated amount of calcium and more neovessels in comparison with control valves [4, 17, 18]. For validation of proteomics results, a separate matching group of patients (*n* = 39) was selected.

**Table 1:** Demographics of the patients examined in the proteomic analysis

	Control	Aortic Stenosis	*P*-value
Patients, n	5	7	
Male, n (%)	5 (100%)	5 (71.4%)	0.46
Bicuspid valve	0	2 (28.6%)	0.47
Age (years), range	43.4 (33.2–53.6)	66.6 ± (53.8–79.3)	0.007
LVEF (SD)	58.0 ± 11.2	56.7 ± 8.8	0.53
DM, n (%)	0	1 (14.3%)	≥0.9
CHD, n (%)	0	3 (42.9%)	0.21
ASO, n (%)	0	1 (14.3%)	≥0.9
Statin use, n (%)	1 (20%)	4 (66.7%)	0.24

*ASO* Peripheral atherosclerosis, *CHD* Coronary heart disease, *DM* Diabetes mellitus, *LVEF* Left ventricle ejection fraction; SD, standard deviation

### Two-dimensional difference gel electrophoresis (2D-DIGE)

The proteins extracted from control (C, *n* = 5) and calcified (AS, *n* = 7) aortic valves were further purified by buffer exchange using an Amicon Ultra ultrafiltration unit with a 10 kDa cutoff (Millipore) and urea buffer (7 M urea, 2 M thiourea, 4% [w/v] CHAPS, 30 mM Tris, pH 8.5) and then the protein samples were sonicated and centrifuged. Protein amounts in the supernatants were determined with a Bradford-based assay according to the manufacturer’s instructions (Roti®-Nanoquant) and the aliquots were stored at − 70 °C. Protein labeling was performed with CyDye DIGE Fluor minimal dyes (GE Healthcare) according to the manufacturer’s protocol using 400 pmol Cy3 (pooled standard) and Cy5 (control, AS, respectively) for 50 μg protein. Proteins were separated as described earlier [19]. In brief, immobilized pH gradient (IPG) strips (pH 3–10 nonlinear, 24 cm, GE Healthcare) were incubated overnight in 650 μl rehydration buffer (7 M urea, 2 M thiourea, 4% [w/v] CHAPS, 130 mM [w/v] DTT, 2%[v/v] carrier ampholytes 3–10, Complete Mini protease inhibitor cocktail [Roche Life Science]). Isoelectric focusing (IEF) after anodic sample cup-loading was carried out with the Multiphor II system (GE Healthcare) under paraffin oil with 67 kVh. SDS-PAGE was performed overnight in polyacrylamide gels (12.5%) with the Ettan DALT II system (GE Healthcare) at 1–2 W per gel in 12 °C. Fluorescence signals were detected with a Typhoon 9400 (GE Healthcare) and 2-D gels analyzed with Delta2D 4.0 (Decodon). Theoretical spot positions were calculated with the Compute pI/Mw tool (http://ca.expasy.org/tools/pi_tool.html). Principal Component Analysis was performed with the Delta2D v4.0 software (Decodon) according to the spot intensities on every gel image.

### Mass spectrometry

For protein identification, additional 2-D gels were run with a higher amount of unlabelled protein (400–600 μg) combined with 50 μg Cy3-labelled internal standard. After detection of the fluorescence signals (see above) and silver staining, labelled and unlabelled protein patterns were matched with the 2-D PAGE image analysis software Melanie 3.0 (GeneBio). Spots with correctly matched centers were excised, digested with trypsin (recombinant; Roche) and prepared for MALDI-TOF mass spectrometry as described previously [19]. The extracted and dried peptides were dissolved in 5 μl alpha-Cyano-3-hydroxycinnamic acid (98%, recrystallized from ethanol-water, 5 mg/ ml in 50% acetonitrile and 0.1% TFA) and 0.5 μl applied onto the sample plate using the dried-droplet method. Proteins were identified from PMF obtained with a VOYAGER-DE™ STR (Applied Biosystems) as described earlier [19]. In general, the clearest peaks (up to 50) visible in the mass spectrum were used to identify proteins with Mascot (http://www.matrixscience.com/) using Swiss-Prot as the corresponding protein database. Search parameters were enzyme: trypsin; modifications: oxidation of Met; missed cleavage: 1; resolution: monoisotopic; ion mode: [M + H]; threshold: 50 ppm. The protein identification was accepted if at least 4 major peaks matched to the protein with the highest Mascot score. In addition, the identification was confirmed by analyzing the induced spot from different gels. During later stages of the project, mass spectra of the tryptic digests were obtained with a UltrafleXtreme MALDI TOF/TOF instrument (Bruker Daltonics) where up to 10 ions from each peptide fingerprint were subjected to the MS/MS measurement. Data were processed with Flexanalyis and Biotools (Bruker) and combined PMF/MS/MS spectra were searched against the NCBI or Swiss-Prot non-redundant protein database using Mascot (Matrix science) with standard search parameters (MS tolerance: 30 ppm, MS/MS tolerance: 0.7 Da, modifications: Carbamidomethyl (Cys) and optional oxidation of Met, up to 1 missed cleavage).

### Protein extraction and Western blot

In the western blot experiments, aortic valve samples were obtained from a separate matching cohort (C, *n* = 19 AS, *n* = 20). The samples were ground in liquid nitrogen, and then homogenized for 10 min in a lysis buffer containing inhibitors. The lysis buffer itself contained 1 M Tris (pH 7.5), 3 M NaCl, 0.25 M EDTA (pH 8.0), 0.1 M EGTA (pH 7.9), 1 mmol/l β-glycerophosphate, 1 mmol/l Na3VO4, 2 mmol/l benzamidine, 1 mmol/l phenylmethylsulfoxide, 50 mmol/l NaF, 1 mmol/l dithiothreitol and 10 μg/ml each of leupeptin, pepstatin, aprotininand and distilled water. The valve tissue samples were homogenized using a MagnaLyser instrument (Roche). After homogenization, the samples were centrifuged for 20 min in 12,500 rpm and + 4 °C and then the supernatant was collected for protein isolation. 5x NEB lysis buffer (100 mM Tris-HCl [pH 7.5], 750 mM.

NaCl, 5 mM EDTA, 5 mM EGTA, 5% Triton X 100, 12 mM sodium pyrophosphate, 5 mM β-glycerophosphate, 5 mM Na_3_VO_4_) was added and mixed following centrifugation for 20 min in 12,500 rpm in + 4 °C. Supernatant containing the total fraction was collected. Western blot was performed using a 1.0 mm, 12% gel with 40 μg of protein/well. The following primary antibodies were used: HSP90α (ADI-SPS-771) and HSP90β (ADI-SPA-844) from Enzo Life Sciences, Protein kinase B (Akt) (#9272), Phospho- Akt (#4056), p38 mitogen activated protein kinase (MAPK) (#9212), phospho-p38 MAPK (#9211), extracellular signal regulated kinase p44/42 MAPK (Erk1/2) (#9102) and Phospho-p44/42 MAPK (pErk1/2) (#9106) from Cell Signaling Technology, Inc., Anti-Annexin II (610,068, BD Transduction Laboratories), and Anti-Galectin 1 (ab25138, Abcam). Anti-mouse-IgG HRP-labeled (GE Healthcare), Anti-rabbit-IgG Peroxidase conjugate (Calbiochem), anti-IgG HRP-linked rabbit (#7074, Cell Signaling Technology, Inc.), and anti-IgG HRP-linked mouse (#7076, Cell Signaling Technology, Inc.) secondary antibodies were used. Data was quantified using the QuantityOne Software (Bio-Rad).

### Histological stainings

The localization of HSP90α and HSP90β in the aortic valve cusps was studied by using immunohistochemical stainings. The aortic valve samples, sent for routine diagnistics, were fixed in buffered formalin solution and embedded in paraffin. Decalcification with EDTA was done if needed. For total valve area and calcified valve area slides were photographed with a Leica DFC420 camera (Wetzlar) and areas were quantified with Image J analysis software. Calcified area to total area was calculated with the following formula: (calcified valve area/ total valve area)*100. Before application of the primary antibodies, the 5-μm-thick sections of valves samples were heated in a microwave oven in citrate buffer, pH 6.0, for 30 min. Rabbit monoclonal antibodies ab133492 at a dilution of 1:2000 (Abcam) for HSP90α and ab32568 at a dilution of 1:300 (Abcam) for HSP90β were used to stain. 3,3′Diaminobenzidine (DAP) was used as the chromogen in immunostaining process. Negative control stainings were carried out by substituting nonimmune rabbit serum for the primary antibodies.

### Molecular network analysis

The up- or down-regulated proteins with their respective expression values were uploaded for processing by the Ingenuity Pathway Analysis (IPA) software (Qiagen). A core analysis was performed with the following parameters: core analysis, reference set user-defined (i.e., only the set of differentially expressed genes by GeneSpring-software mapped to the IPA database), direct and indirect relationships included, confidence = experimentally observed. Then, the IPA-software was used to generate a molecular network showing the interrelationships between up- or down-regulated proteins as previously described [20], based on the information contained in the Ingenuity Pathways Knowledge database.

### Statistical analysis

The results are expressed as mean with standard deviation (SD) unless otherwise stated. Continuous variables were analyzed by using Student’s t-test, semi-continuous variables using Mann-Whitney U-test and Fisher’s exact test for categorical variables. Analyses were performed using SPSS for Windows (IBM Corp. Released 2018. IBM SPSS Statistics for Windows, Version 25.0). Correlations were detected with linear regression model in Graphpad Prism 5. *P* < 0.05 was considered statistically significant.

## Results

### Proteomic analysis of aortic valve calcification

**Fig. 1:**
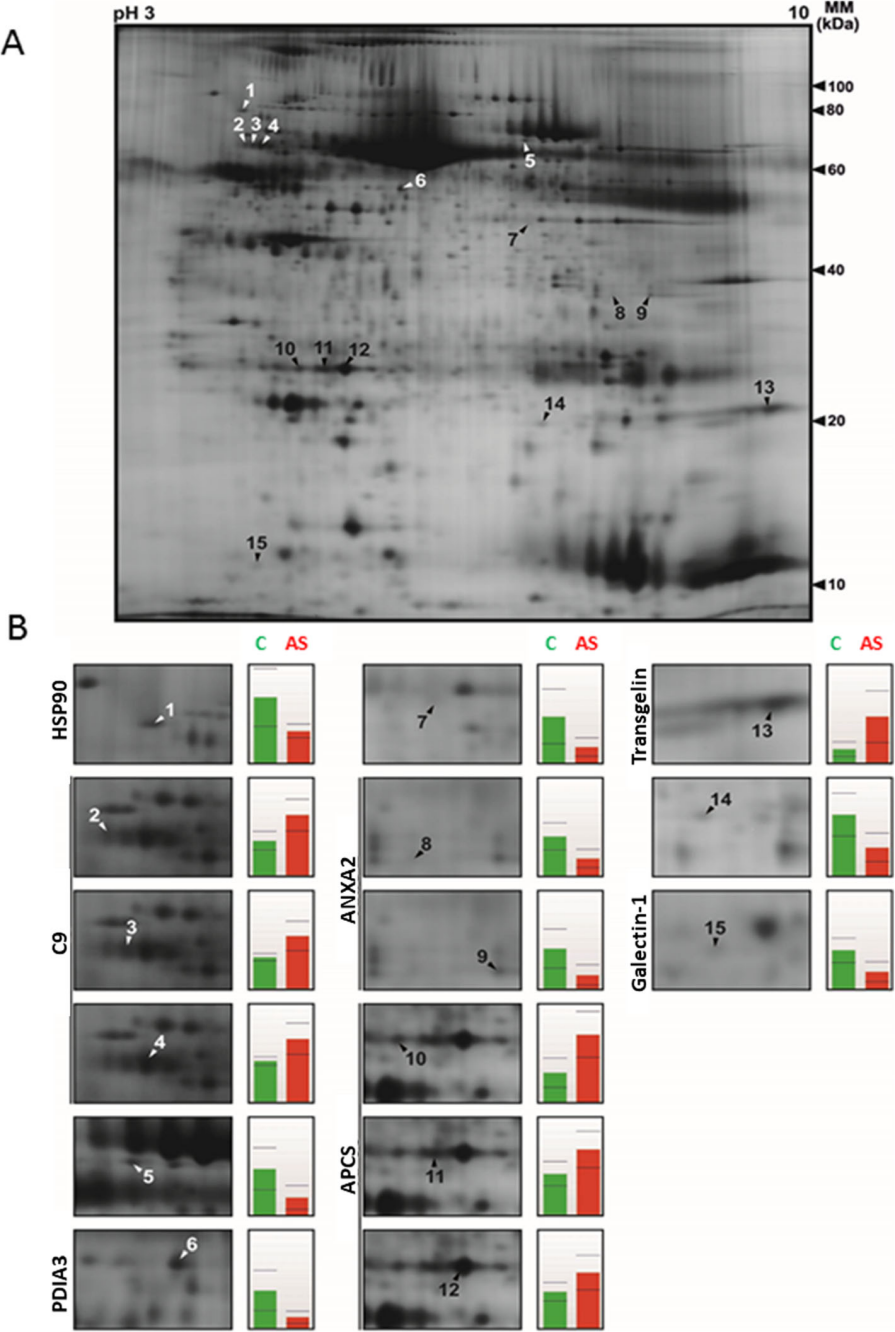
CAVD-related proteomic changes in human aortic valves. **a** Representative 2-D gel of calcified aortic valve is shown. Proteins (50 μg) were labelled with minimal DIGE and separated by IEF (pH 3–10 NL) and SDS-PAGE. **b** The positions of the changed spots as well as the expression profiles indicating the detected protein levels in control (C) and stenotic (AS) aortic valves are specified. HSP90, heat-shock protein 90; C9, complement 9; PDIA3, protein disulfide isomerase A3; ANXA2, annexin 2; serum amyloid P-component, APCS

The proteomic analysis, based on the minimal DIGE, identified a total of 15 differentially abundant protein spots in stenotic valves as compared to control valves according to the selection criteria (fold change ≥1.5 and *P* ≤ 0.05). A typical 2D gel representing calcified aortic valve proteins is shown in Fig. 1. Further, mass spectrometry analyses identified seven proteins within 12 spots (Table 2) which had undergone significant upregulation i.e. complement 9, serum amyloid P-component (APCS) and transgelin (1.7-, 2.3- and 3.5-fold, respectively, *P* < 0.05) as well as downregulation of heat shock protein HSP90 (α/β; genes HSP90AA1/AB1), protein disulfide isomerase A3 (PDIA3), annexin A2 (ANXA2) and galectin-1 (2.1-, 3.5-, 2.2- and 2.2-fold, respectively, *P* < 0.05) in stenotic valves (Fig. 1, Table 2).

**Table 2:** Up- and down-regulated proteins as determined by 2D-DIGE in calcified valves as compared to control valves

				Comparison Control vs. calcified valves^a^			Protein identification^c^	
Spot	Protein	UniProt	Description	Ratio	P-value	Theoretical pI/MW (kDa)^b^	Score (MS, MSMS)	SC (P) (MS, MSMS)
1	HSP90α/β	P07900/ P08238	Heat shock protein HSP90AA1/AB1	− 2.05	0.022	AA1: 1: 4.94/84.7	113.0, −	39(18),-
						2: 5.07/98.2		
						AB1: 4.96/83.3		
2	C9	P02748	Complement 9	1.74	0.014	5.43/63.2 (5.42/60.9)	83.2, −	21(14),-
3				1.68	0.016	(C9a: 4.59/27.8)	88.5, −	18(13),-
4				1.55	0.018	(C9b: 8.63/33.2)	-, 72.9	-, 5(2)
6	PDIA3	P30101	Protein disulfide-isomerase A3	−3.35	0.016	5.98/56.8 (5.61/54.3)	162.0,-	46.8(21),-
8	ANXA2	P07355	Annexin A2	−2.22	0.012	1: 7.57/38.6 (7.56/38.5)	71.1,-	21(5),-
9				− 2.95	0.019	2: 8.53/40.4 (8.54/40.3)	199*,-	33(13),-
10	APCS	P02743	Serum amyloid P-component	2.26	0.014	6.10/25.4 (6.12/23.3)	75*, −	20(4),-
11				1.59	0.039		80*,-	20(5),-
12				1.51	0.046		82*,-	25(5),-
13	TAGLN	Q01995	Transgelin	3.45	0.032	8.87/22.6 (8.88/22.5)	176.00,-	76(27),-
15	LGALS1	P09382	Galectin-1	−2.19	0.012	5.30/14.7 (5.30/14.6)	92.4, 64.8	57(7), 44(2)

^a^The ratio represents the change of the mean normalized volumes. Statistical significance is shown with the t-test (*P* < 0.05). Ratio and t-test values of the unidentified spots were as follows: spot 5 (−2.60, 0.0200), spot 7 (− 2.91, 0.0311) and spot 14 (− 2.12, 0.0159)

^b^The theoretical expected spot position in the gel according to the full or matured (in brackets) protein sequence is shown. If the protein exists in different isoforms, then the specific number of the isoform is likewise indicated

^c^The protein identification shows the MS and/or MSMS scores based on analysis with the UltrafleXtreme MALDI TOF/TOF instrument or measurements with the VOYAGER-DE™ STR (*). In addition, the sequence coverage (SC) and number of matched peptides (P) are shown

**Table 3:** Full annotation of genes illustrated in Fig. 4

Symbol	Entrez Gene Name	Location	Family	Entrez Gene
Akt	AKT Serine/Threonine Kinase	Cytoplasm	group	
ALPK1	Alpha kinase 1	Other	kinase	80,216
ANXA2	Annexin A2	Plasma Membrane	other	302
APCS	Amyloid P component, serum	Extracellular Space	other	325
ARMC5	Armadillo repeat containing 5	Cytoplasm	other	79,798
C20orf194	Chromosome 20 open reading frame 194	Nucleus	other	25,943
C9	Complement C9	Extracellular Space	other	735
Ck2	Casein Kinase II	Cytoplasm	complex	
DCLK2	Doublecortin like kinase 2	Cytoplasm	kinase	166,614
DDX59	DEAD-box helicase 59	Other	enzyme	83,479
DMRTA1	DMRT like family A1	Nucleus	transcription regulator	63,951
ERK	Extracellular Signal-Regulated Kinase 1/2	Other	group	
G2E3	G2/M-phase specific E3 ubiquitin protein ligase	Cytoplasm	enzyme	55,632
GRK7	G protein-coupled receptor kinase 7	Cytoplasm	kinase	131,890
HSP90	Heat shock protein 90	Cytoplasm	group	
HSP90AA1	Heat shock protein 90 alpha family class A member 1	Cytoplasm	enzyme	3320
HSP90AB1	Heat shock protein 90 alpha family class B member 1	Cytoplasm	enzyme	3326
IL6	Interleukin 6	Extracellular Space	cytokine	3569
INSRR	Insulin receptor related receptor	Plasma Membrane	kinase	3645
KCTD8	Potassium channel tetramerization domain containing 8	Other	other	386,617
LGALS1	Galectin 1	Extracellular Space	other	3956
MAP 3 K15	Mitogen-activated protein kinase kinase kinase 15	Other	other	389,840
MYLK4	Myosin light chain kinase family member 4	Cytoplasm	kinase	340,156
MYO3B	Myosin IIIB	Plasma Membrane	kinase	140,469
P38 MAPK	Mitogen-Activated Protein Kinase P38 Alpha	Cytoplasm	group	1432
PDIA3	Protein disulfide isomerase family A member 3	Cytoplasm	peptidase	2923
PSKH1	Protein serine kinase H1	Nucleus	kinase	5681
RPS6KL1	Ribosomal protein S6 kinase like 1	Other	kinase	83,694
STK32B	Serine/threonine kinase 32B	Other	kinase	55,351
TAGLN	Transgelin	Cytoplasm	other	6876
TNF	Tumor necrosis factor	Extracellular Space	cytokine	7124
TSSK2	Testis specific serine kinase 2	Cytoplasm	kinase	23,617
ZBED4	Zinc finger BED-type containing 4	Nucleus	transcription regulator	9889
ZNF215	Zinc finger protein 215	Nucleus	transcription regulator	7762

### Confirmation of proteomics results

**Fig. 2:**
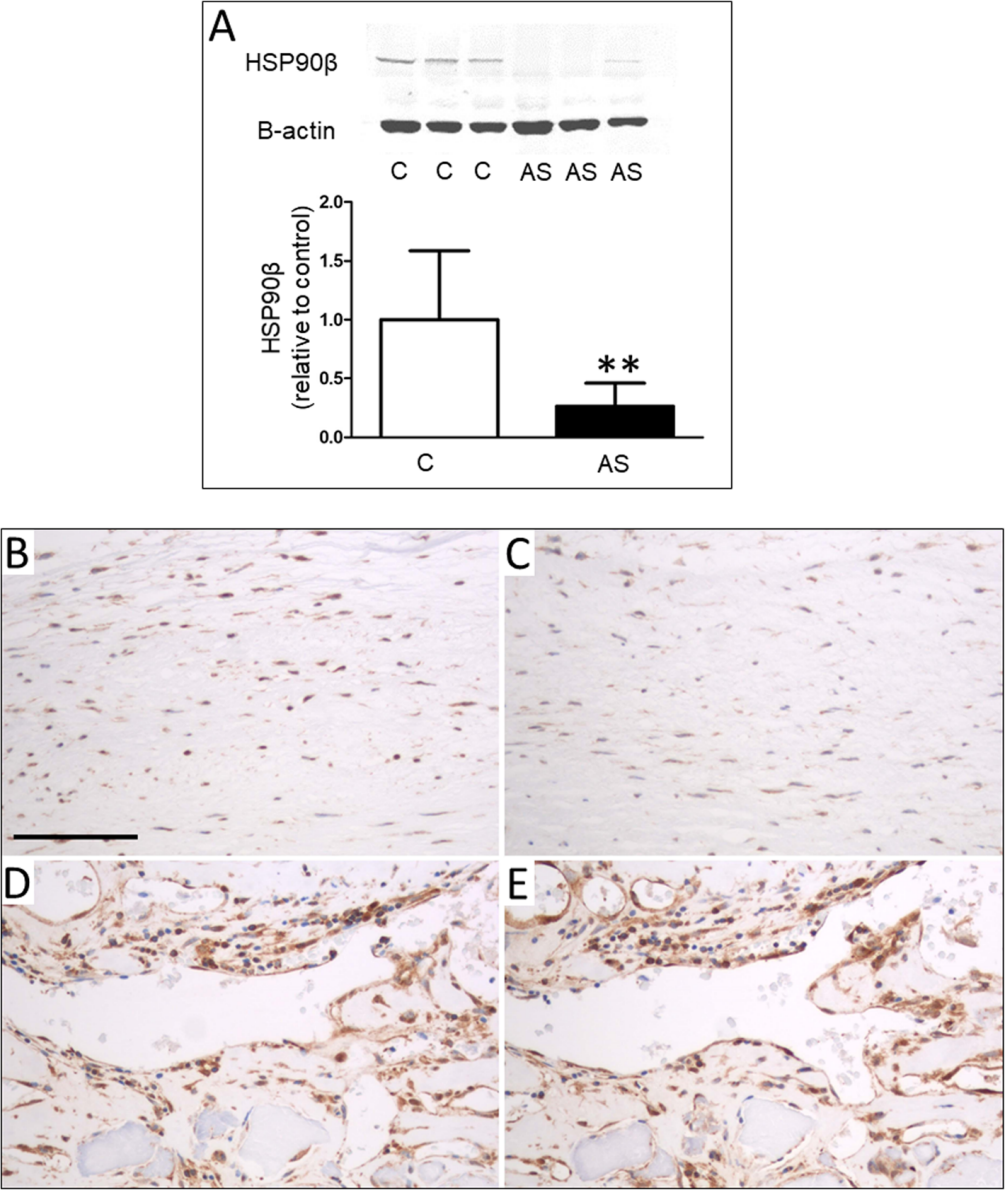
HSP90 expression in aortic valves. **a** Western blot analysis revealed decreased HSP90β protein levels in stenotic valves (AS) when compared to control valves (C). Results are mean ± SD, ** = *P* < 0.01. Representative Western blots are shown. Immunohistochemical stainings against HSP90α (**b**, **d**) and HSP90β (**c**, **e**) in aortic valves. VICs in aortic valve displayed cytoplasmic positivity for HSP90α (**b**) and HSP90β (**c**) stainings. Representative examples of adjacent sections of the same area of a control valve. Also the the endothelium was strongly positive for HSP90α (**d**) and HSP90β (**e**). Representative examples of adjacent sections of the same area of neovasculature in calcified valves. There was also a wide positive reaction in valve interstitial cells (VICs) and patchy positivity in inflammatory cells, mainly small lymphocytes. All pictures are at the same scale, scale bar depicts 100 μm

**Fig. 3:**
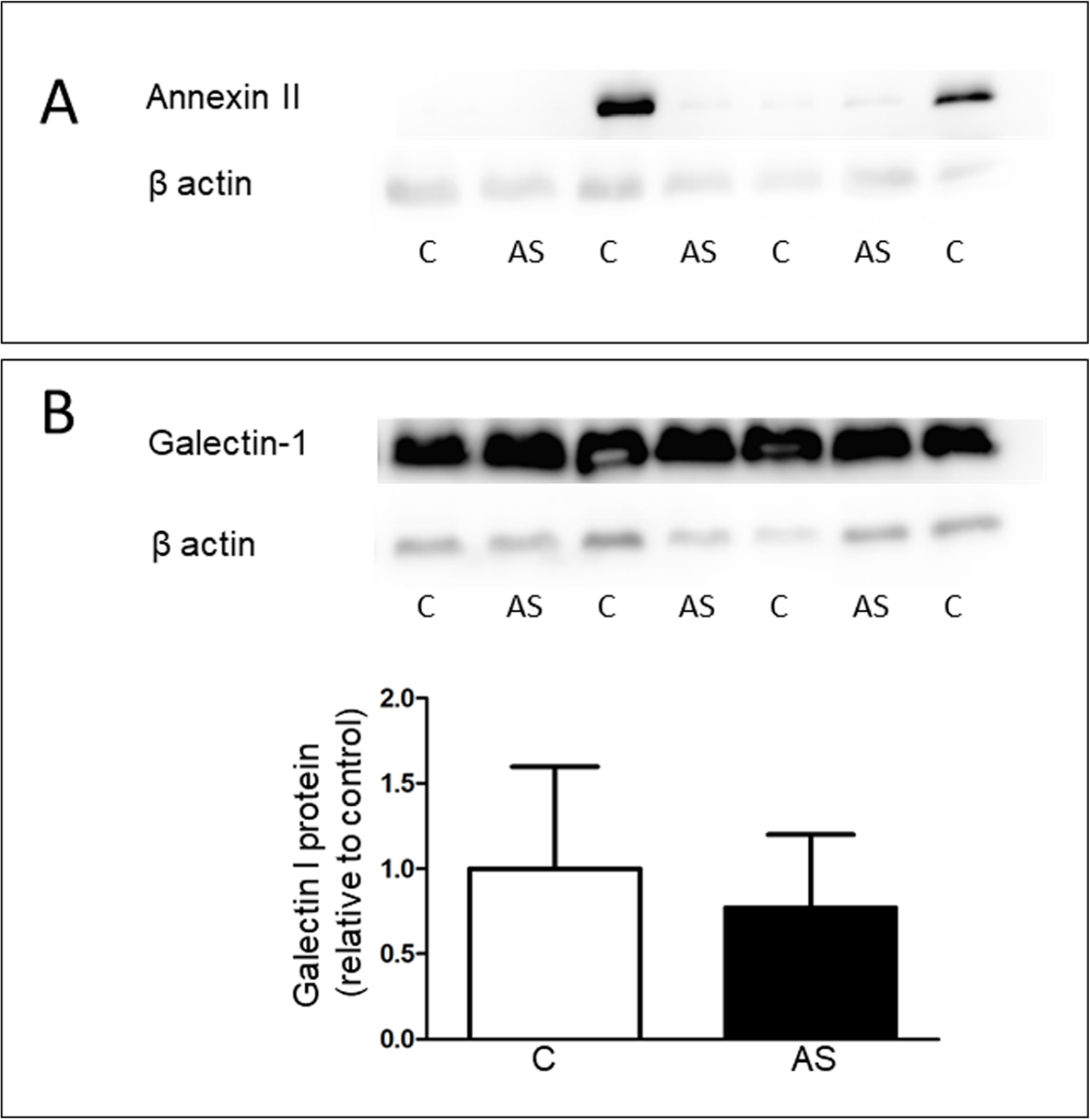
Protein expression of annexin II and galectin-1 in aortic valves. Western blot analysis showing (**a**) annexin II and (**b**) galectin-1 in stenotic (AS) and control valves (C). Representative Western blots are shown. Results are mean ± SD

Western blot was used to validate the the proteomic results of HSP90, ANXA2 and galectin-1. The candidate proteins were selected based on their unknown role in CAVD. A marked downregulation of HSP90β protein levels was detected in stenotic valves compared to controls (Fig. 2a-b), whereas no change in HSP90α protein levels was observed (data not shown). Considerable interindividual variability was seen in the levels of ANXA2 protein, since it was highly expressed in only two out of three control samples (Fig. 3c). There was no significant difference in galectin-1 protein levels between stenotic and control levels (Fig. 3a-b).

To test if valvular anatomy impact on our data, we performed an unsupervised clustering analysis (principal component analysis, PCA) on the raw proteomic data (Additional file 1: Figure S1). We hypothesized that if valvular anatomy would affect protein expression profile, bicuspid valves should cluster together and distinctly from tricuspid valves. However, we find no evidence of such clustering and in fact, the clearest distinction emerges between control, and AS.

We correlated HSP90 expression levels both with age and calcification of the valves (expressed as proportion of calcified area in aortic valve cusps to total aortic valve). As shown in Additional file 2: Figure S2A-B, the HSP90β protein levels correlated with age of the patients (*P* < 0.01) and calcification of the valves (*P* < 0.05). In addition, the valvular calcification correlated with the age (*P* < 0.01) (Additional file 2: Figure S2C).

In the immunohistochemical stainings, the localization of HSP90α and HSP90β was virtually identical (Fig. 2b-e). VICs in both normal and calcified valves were positive. In addition, the endothelium of neovasculature was widely positively stained, whereas in the surface endothelium, the positive reaction was more patchy. Furthermore, most of the inflammatory cells, mainly lymphocytes, were also positively stained.

### Identification of the molecular network between up- and down-regulated proteins

**Fig. 4:**
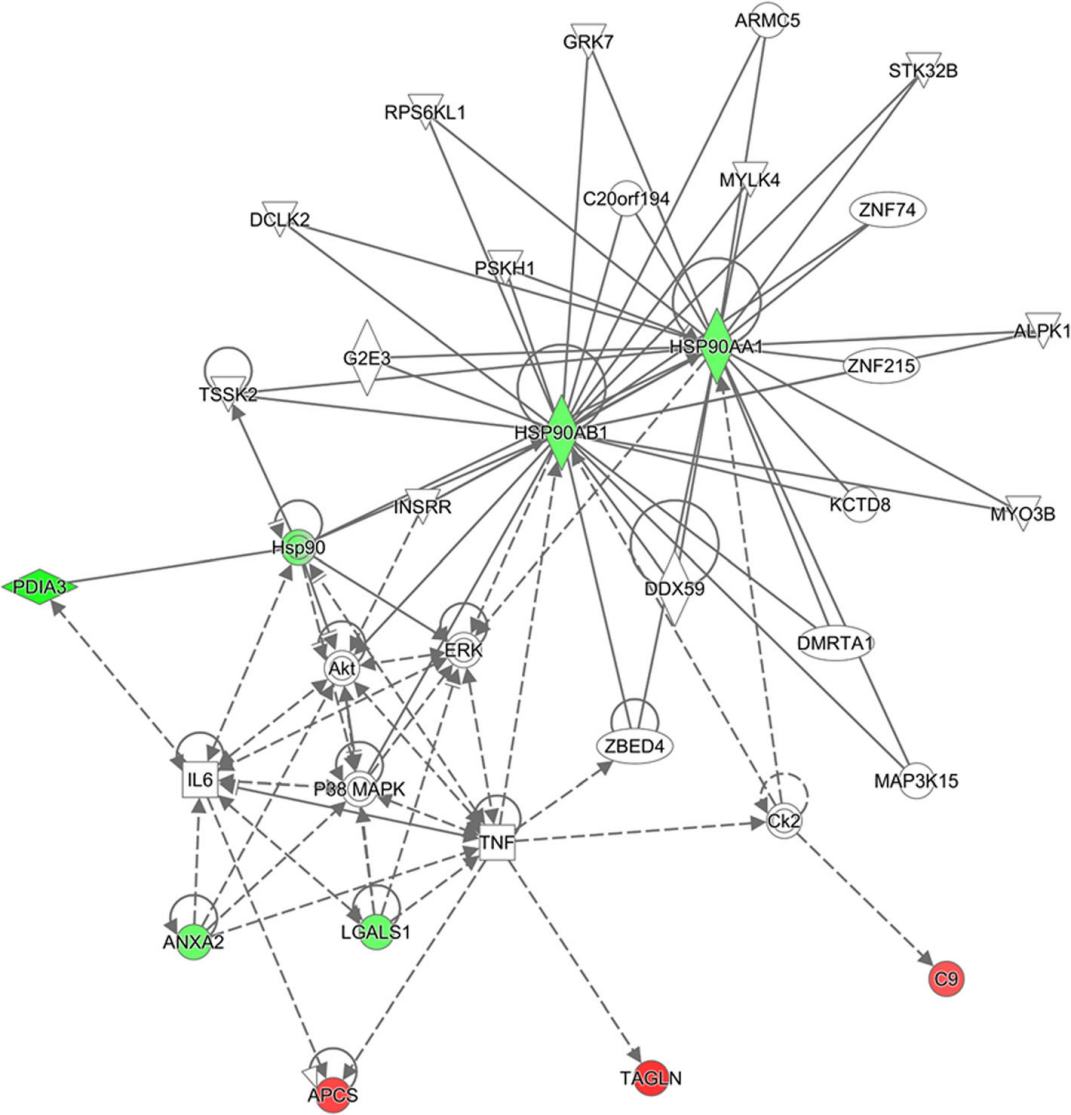
The molecular network of differentially expressed proteins in CAVD generated by Ingenuity Pathway Analysis. The Ingenuity Pathway Analysis (IPA) Core Analysis-based network displays interactions between proteins that were differentially expressed in stenotic valves as compared to control valves. Up- and down-regulated proteins are in red and green, respectively. Molecules not marked with a color were not altered in the data set but they are possible connections suggested by IPA. Molecules are represented with various shapes that represent the functional class of the gene product. A solid line represents direct interactions and a dashed line represents an indirect interaction. The full names of the molecules are given in Table 3

An IPA-analysis was used to determine the biological relationships among the differentially expressed proteins. The main molecular network exhibiting expression changes based on Fisher’s exact test is shown in Fig. 4.

**Fig. 5:**
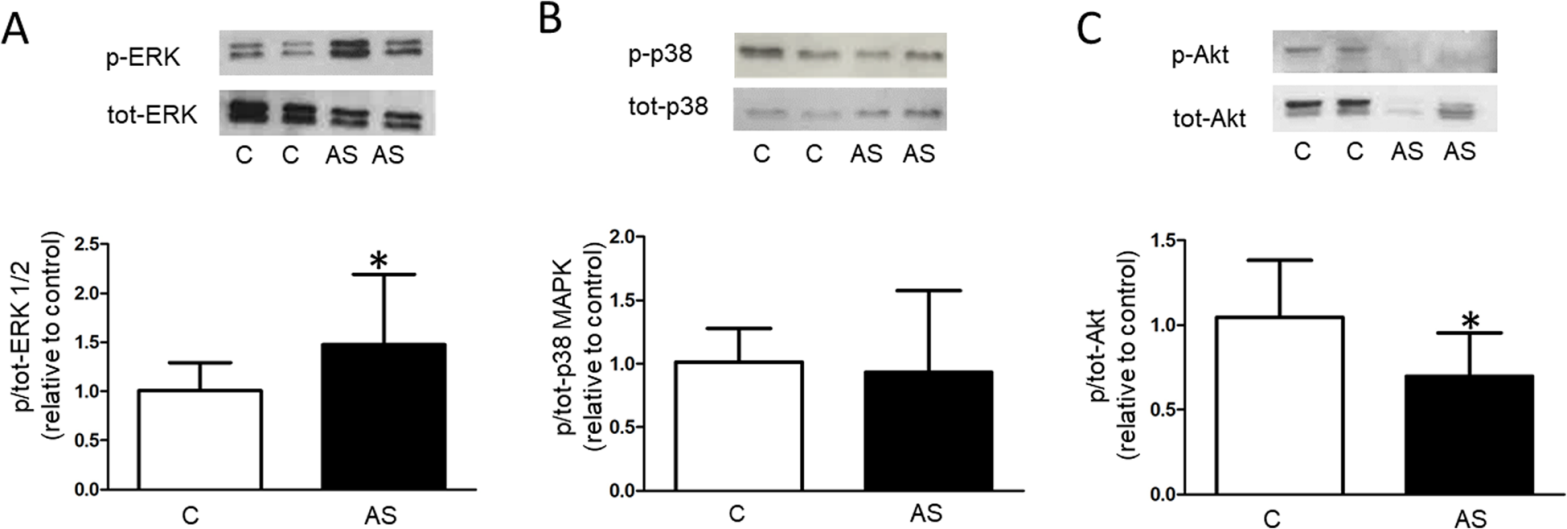
Phosphorylation of protein kinases in stenotic (AS) and control (C) valves. Western blot analysis of A) Extracellular signal regulated kinase 1/2 (ERK) 1/2), B) p38 Mitogen activated protein kinase (p38 MAPK) and C) Protein kinase B (Akt). The results in bar graphs are mean ± SD and expressed as the ratio of the phosphorylated protein kinase to total protein kinase. Representative Western blots are shown.*P < 0.05

Among the novel putative interactions suggested by IPA, HSP90 was linked to Akt and ERK, and further to p38 MAPK (Fig. 4). Therefore, we conducted Western blot analyses to evaluate the activation of Akt, ERK and p38 MAPK kinases in control and stenotic valves. The ratio of phosphorylated ERK to total ERK was increased (1.5-fold, *P* < 0.05) whereas the ratio of phosphorylated Akt to total Akt was reduced (0.7-fold, *P* < 0.05) suggesting that the Akt and ERK pathways were disturbed in the stenotic valves (Fig. 5a,c). Instead, there was no change in the phosphorylation pattern of p38 MAPK in stenotic valves (Fig. 5b).

## Discussion

Here we describe a 2D-DIGE-determined proteomic profile associated with CAVD. We detected a distinct upregulation of APCS, C9 and transgelin as well as a downregulation of HSP90, PDIA3, ANXA2 and galectin-1 proteins in calcified valves in comparison to control valves. We confirmed this decrease in HSP90β protein levels in calcified valves by performing a Western blot analysis and then conducted an IPA analysis to predict HSP90 interactions in CAVD.

This is the first study revealing a decreased expression of HSP90 in calcified aortic valves. HSP90 is a molecular chaperone and a member of a large HSP family. Previously, a decreased expression of HSP27 in calcified valves has been reported in the proteomic study conducted by Martin-Rojas et al. [16]. In addition, several HSP family members are linked to the pathophysiology of atherosclerosis in which they have pro-inflammatory effects and regulate endothelial function (reviewed in [21]). In the endothelium, HSP90 is part of a complex with endothelial nitric oxide synthase ((eNOS)/HSP90) [22–24]. The dissociation of HSP90 causes uncoupling of eNOS, leading to the production of reactive oxygen species (ROS) and endothelial dysfunction [23]. This process might be initiated by pro-inflammatory lipids [23, 24]. Interestingly, uncoupling of NOS and the increased level of oxidative stress have also been reported in calcified stenotic aortic valves [25]. In our study, HSP90 positivity was seen in the surface endothelium of the valves, and in the endothelium of the neovasculature of the calcified valves. Our findings are similar to those of Martín-Rojas et al. [16], who reported decreased HSP27 protein levels in the endothelium layer of stenotic valves [16]. In summary, the expression of HSP90 in the endothelium supports the idea that HSP90 has a role in regulating endothelial function in the progression of aortic valve calcification.

HSP90 was expressed in inflammatory cells that were mainly lymphocytes. In atherosclerosis, HSP90 is overexpressed in inflammatory sites of human atherosclerotic plaques [26, 27]. Furthermore, several studies have reported that inhibition of HSP90 can exert atheroprotective effects (decreased plaque size and reduced inflammatory responses) [27, 28] and less oxidative stress [29]. In CAVD, the amount of inflammation decreases when the disease progresses and acquires its more calcific phenotype (reviewed in [2]). If the role of HSP90 is related to the propagation of inflammation, it may have a different function in a heavily calcific environment as was present in these stenotic valves. However, further studies should focus on HSP90’s role in calcific VICs.

HSP90 mediates an ATP-dependent folding of its target proteins that are involved in many diverse cellular processes ranging from intracellular transportation to signal transduction (for general reviews on HSP90 biology, see [30]. The IPA analysis predicted that downregulation of HSP90 would be linked with Akt, p38 MAPK and ERK signalling pathways. We observed an increased activation of ERK1/2 and reduced activation of Akt-kinase in calcified valves whereas there was no change in the phosphorylation of p38 MAPK. Previously, Akt and ERK have been shown to mediate leptin induced osteoblast differentiation [31]. In addition, activation of Akt has been demonstrated to regulate calcium deposition [32] and ROS-induced expression of RUNX2 in human VICs [33]. Furthermore, a study with cultured porcine VICs revealed that ERK inhibition reduced gene expression of myofibroblastic and osteoblastic markers [34]. In our study, Akt and ERK exhibited distinctive phosphorylation patterns suggesting differential regulation of these signalling pathways in the heavily calcified valves.

In agreement with the previous proteomics studies of CAVD [10], [12], [16], we identified increased protein expression of the C9 component of the complement system and APCS. Upregulation of C9 is supported by the data by Helske et al. [35] who were the first investigators to show an activation of the complement system in stenotic valves. APCS, also known as pentraxin-2, is involved in amyloidosis but it is also present in human atherosclerotic lesions [36]. It exerts anti-inflammatory and antifibrotic properties e.g. inhibiting monocyte differentiation into proinflammatory macrophages [37]. APCS has also been implicated in several cardiovascular pathologies (reviewed in [38–40]), and the role of this protein in CAVD should be investigated in detail. Our finding of decreased ANXA2 protein levels in calcified valves confirms the similar proteomic profiling findings of Matsumoto et al. [10]. In addition, ANXA1 and ANXA3 have been previously detected in calcified regions of aortic valves [10, 12]. Furthermore, Cui et al. [41] detected an up-regulation of annexins I, II, III, IV, V, VI, VII, and XI in calcifying VIC-derived matrix vesicles, highlighting the significance of the annexins in the calcification process. However, in contrast to a previous proteomic study [16], we observed increased transgelin (SM22) levels in stenotic valves. Since in both studies, transgelin was detected at different positions in the 2D gel, this discrepancy might be explained by the presence of different transgelin variants.

A major limitation of our work is small number of valves in our proteomic profiling study. This limited sample size raises issue that results do not represent heterogeneity of aortic stenosis patients. This might be the reason why we were not able to confirm with the Western blots the results of the proteomic data on Annexin II and galectin-1. Consequently, the generalizability of these results have to be confirmed in larger sample population. However, it is noteworthy that despite limited number patients, we also noticed increased expressions of CP9 component, APCS and ANXA2, in agreement with previous proteomic studies.

In our analysis, HSP90 expression correlated both with age and calcification of the valves. Since aortic valve calcification correlated with age of the patients, these correlations can be explained to be due the fact that aortic valve calcification is most prevalent in the elderly [42]. Thus, we cannot rule out that the changes in HSP90 protein expression are due to the aging and not aortic valve calcification. Furthermore, we did not confirm all the proteomic profiling results with another method. Finally, all the HSP90 associations are only predicted; the experimental validation of HSP90 interaction with Akt and ERK signaling pathways in aortic valve calcification will have to be the subject of some future work.

## Conclusion

Our proteomic analysis identified seven dysregulated proteins in calcified valves when compared to control valves. These proteins may have roles in regulating processes associated with the pathogenesis of CAVD such as the immune response and calcification. Overall, our findings suggest novel insights into the mechanisms of aortic valve calcification and HSP90 may be a central signaling molecule in aortic valve calcification.

## Supplementary information

**Additional file 1: Figure S1.**. An unsupervised clustering analysis (principal component analysis, PCA) on the raw proteomic data was performed to test if valvular anatomy impact on our data. PCA shows the clearest distinction emerges between control, and aortic stenotic group (diseased). Bicuspid valves (1574 and 1575, circulated with red colour) do not cluster together and distinctly from tricuspid valves.

**Additional file 2: Figure S2.** Correlations between heat-shock protein 90 (HSP90), age of the patients and calcification of the valves. A) HSP90 correlated with the age of the patients (y). Additionally, B) calcium area of the total valve area (%) correlated with the relative expression of HSP90 C) The age of the patients correlated with calcium area of the total valve area.

## Data Availability

The dataset supporting the conclusions of this article is included within the article. The raw data used and/or analysed during the current study are available from the corresponding author on reasonable request.

## Abbreviations

2D-DIGE: Two-dimensional difference gel electrophoresis
Akt: Protein kinase B
ANXA2: Annexin A2
APCS: Serum amyloid P-component
AS: Aortic stenosis
BMP2: Bone morphogenic protein 2
C9: Complement 9
CAVD: Calcific aortic valve disease
eNOS: Endothelial nitric oxide synthase
ERK: Extracellular signal regulated kinase
HSP90: Heat shock protein 90
MAPK: Mitogen activated protein kinase
PDIA3: Protein disulfide isomerase A3
ROS: Reactive oxygen species
RUNX2: Runt-related transcription factor 2
VICs: Valve interstitial cells
