# Supplementary material for: Heat shock protein 90 is downregulated in calcific aortic valve disease
Source: BMC Cardiovasc Disord. 2019 Dec 19;19:306. doi: 10.1186/s12872-019-01294-2 (PMC6923932; doi:10.1186/s12872-019-01294-2)
Supplement: Supplementary file 1 — Additional file 1: Figure S1.. An unsupervised clustering analysis (principal component analysis, PCA) on the raw proteomic data was performed to test if valvular anatomy impact on our data. PCA shows the clearest distinction emerges between control, and aortic stenotic group (diseased). Bicuspid valves (1574 and 1575, circulated with red colour) do not cluster together and distinctly from tricuspid valves. [file 12872_2019_1294_MOESM1_ESM.pdf]

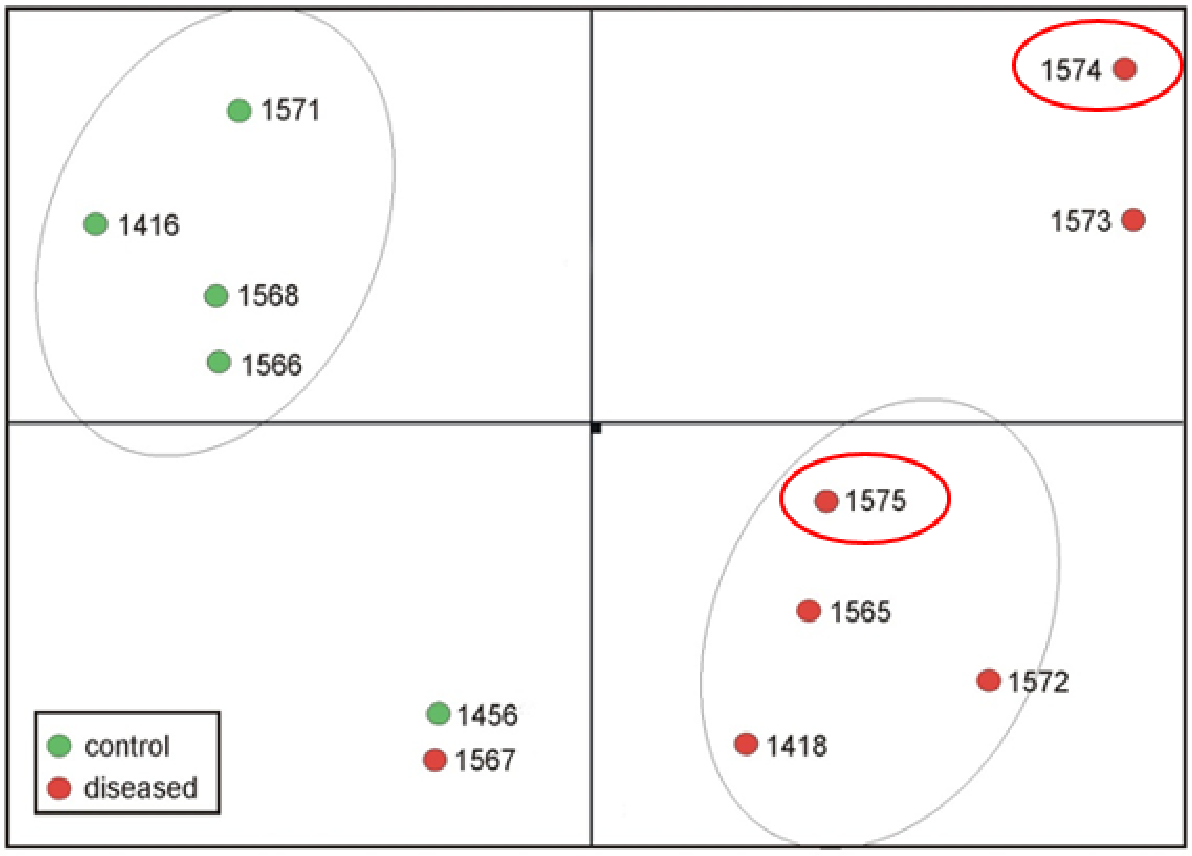

Supplemental Figure 1. An unsupervised clustering analysis (principal component analysis, PCA) on the raw proteomic data was performed to test if valvular anatomy impact on our data. PCA shows the clearest distinction emerges between control, and aortic stenotic group (diseased). Bicuspid valves (1574 and 1575, circulated with red colour) do not cluster together and distinctly from tricuspid valves.
