# Supplementary material for: Heat shock protein 90 is downregulated in calcific aortic valve disease
Source: BMC Cardiovasc Disord. 2019 Dec 19;19:306. doi: 10.1186/s12872-019-01294-2 (PMC6923932; doi:10.1186/s12872-019-01294-2)
Supplement: Supplementary file 2 — Additional file 2: Figure S2. Correlations between heat-shock protein 90 (HSP90), age of the patients and calcification of the valves. A) HSP90 correlated with the age of the patients (y). Additionally, B) calcium area of the total valve area (%) correlated with the relative expression of HSP90 C) The age of the patients correlated with calcium area of the total valve area. [file 12872_2019_1294_MOESM2_ESM.pdf]

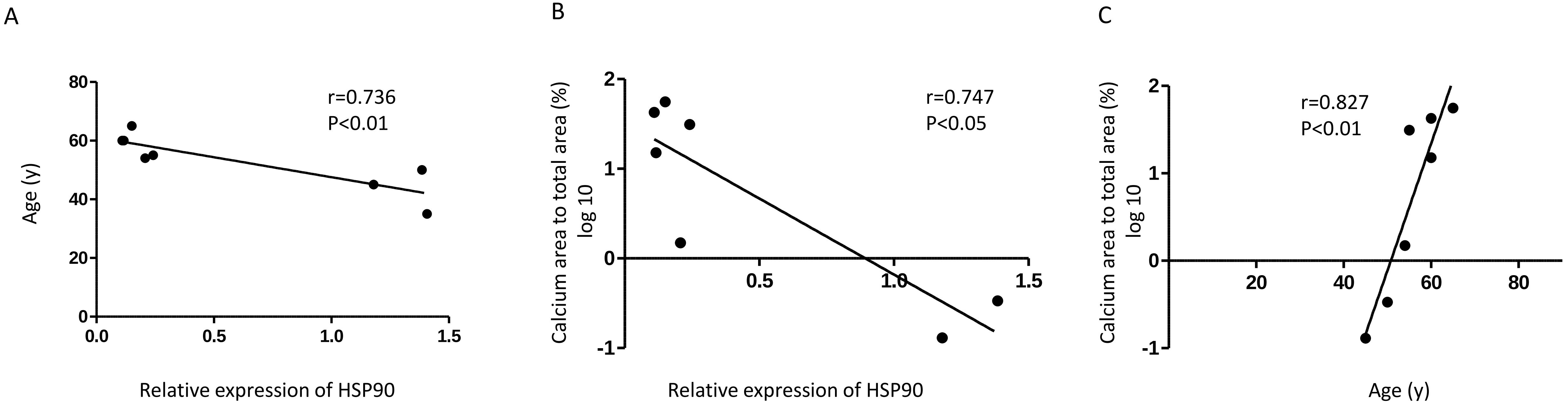

Supplemental figure 2. Correlations between heat-shock protein 90 (HSP90), age of the patients and calcification of the valves. A) HSP90 correlated with the age of the patients (y). Additionally, B) calcium area of the total valve area (%) correlated with the relative expression of HSP90 C) The age of the patients correlated with calcium area of the total valve area.
